# The Association of Fat-Mass-and Obesity-Associated Gene Polymorphism (rs9939609) With Colorectal Cancer: A Case-Control Study

**DOI:** 10.3389/fonc.2021.732515

**Published:** 2021-09-28

**Authors:** Maryam Gholamalizadeh, Mohammad Esmail Akbari, Saeid Doaei, Sayed Hossein Davoodi, Bojlul Bahar, Ghasem Azizi Tabesh, Hossein Sadeghi, Melika Razavi Hashemi, Elham Kheyrani, Samira Rastgoo, Azadeh Hajipour, Zahra Aslany, Reza Mirfakhraie, Alireza Mosavi Jarrahi

**Affiliations:** ^1^ Student Research Committee, Cancer Research Center, Shahid Beheshti University of Medical Sciences, Tehran, Iran; ^2^ Cancer Research Center, Shahid Beheshti University of Medical Sciences, Tehran, Iran; ^3^ Research Center of Health and Environment, School of Health, Guilan University of Medical Sciences, Rasht, Iran; ^4^ Departments of Clinical Nutrition and Dietetics, Faculty of Nutrition and Food Technology, National Nutrition and Food Technology Research Institute, Shahid Beheshti University of Medical Sciences, Tehran, Iran; ^5^ Nutrition Sciences and Applied Food Safety Studies, Research Centre for Global Development, School of Sport & Health Sciences, University of Central Lancashire, Preston, United Kingdom; ^6^ Department of Community Nutrition, School of Nutritional Sciences and Dietetics, Tehran University of Medical Sciences, Tehran, Iran; ^7^ Genomic Research Center, Shahid Beheshti University of Medical Sciences, Tehran, Iran; ^8^ Department of Pathology, Firoozgar General Hospital, Iran University of Medical Sciences, Tehran, Iran; ^9^ Taban Medical Genetic Laboratory, Tehran, Iran; ^10^ School of Health, Qazvin University of Medical Sciences, Qazvin, Iran; ^11^ School of Medicine, Shahid Beheshti University of Medical Sciences, Tehran, Iran

**Keywords:** colorectal cancer, fat-mass- and obesity-associated (FTO) gene, polymorphism, genotype, colon cancer

## Abstract

**Background and Aim:**

The association between the rs9939609 polymorphism of fat mass and obesity-associated gene (FTO) and risk of colorectal cancer is controversial. This study aims to evaluate the relationship between FTO rs9939609 polymorphism and colorectal cancer (CRC) in Iranian people.

**Methods:**

A case-control study was conducted on 125 patients with CRC and 250 healthy subjects in Tehran, Iran. Demographic data and blood samples were collected from all participants. Genotyping of rs9939609 polymorphism was performed by the tetra-primer amplification refractory mutation system-polymerase chain reaction (T-ARMS-PCR) method.

**Results:**

The occurrence of AA genotype of FTO rs9939609 polymorphism in the colorectal cancer patients was significantly higher compared to that of healthy subjects (16.4 *vs.* 2.9%, respectively, P=0.02). The association between the frequency of risk allele of the FTO polymorphism and CRC (B=1.67, P=0.042) remained significant after adjustment for age. Further adjustment for gender (model 2) and marital status (model 3) did not change this result (B=1.67, P= 0.042 and B=1.67, P=0.043, respectively). The results remained significant after additional adjustment for ethnicity (B=1.57, P= 0.047).

**Conclusion:**

We found a positive association between the A allele of the rs9939609 polymorphism and CRC. Future studies are required to identify the underlying mechanisms.

## Introduction

Colorectal cancer (CRC), also known as bowel cancer, colon cancer, rectal cancer, is characterized by the abnormal growth of tissue in the large intestine leading to cancer (1). With 1–2 million new cases and 700,000 deaths every year, CRC is one of the most common cancers worldwide (2). CRC is among the top three cancers most diagnosed, with an incidence rate of 11% of all cancers. CRC is more prevalent among men than women (3). While the incidence of CRC is high in developed nations, there are rapid increases in cases in developed countries, including Iran. Based on a screening program completed in 2018, 33 Iranians per 100,000 have CRC (4). There has been a year-to-year increase in the overall risk of developing this cancer among the Iranians (5).

The pathophysiology leading to CRC development includes a complex interaction of genotype and environment (lifestyle) factors. The lifestyle factors include excessive indulgence of highly processed and Westernized dietary habits; consumption of highly refined sugars, animal fats; low consumption of fruits and vegetables; consumption of tobacco and alcohol; and low physical activities (6–9). Several recent studies identified obesity as an independent risk factor for CRC across all age groups, further suggesting the role of lifestyle factors in CRC (10). The body mass index (BMI) greater than 30 kg/m^2^ is an independent risk factor of CRC. Genetic factors are estimated to account for 40–70% of obesity causes, and therefore, identifying the genetic loci linked to the development of obesity and CRC has distinct advantages. Genetic determinants/markers are independent of the effect of the environmental/lifestyle factors; once the risk allele is identified, the marker can be measured precisely. Genetic markers are not affected by disease stage and may represent lifelong risk factors of the disease (11).

Several GWAS studies suggested that CRC has a strong genetic basis. To date, more than 50 unique genetic loci, many with a known biochemical basis, are mapped to have an association with the incidence of CRC across different ethnicity (12). Fat-mass- and obesity-associated (FTO) gene codes a polypeptide with 505 amino acids and is a common genetic determinant for obesity and cancer (13). Human FTO protein is expressed in the brain, skeletal muscle, and adipose tissue (8). The expression of FTO within the arcuate nucleus of the hypothalamus is essential for regulating appetite and energy metabolism (14). An excessive expression of FTO increases food intake, thereby leading to a positive energy balance linked to obesity. Hence, FTO expression is suspected to be a risk factor for several cancers as it could result in poorer prognosis and potentially affect therapeutic efficacy (10). Several studies have reported a strong association of the FTO polymorphisms with the risk of developing obesity (9). Recent epidemiological studies also indicated that FTO variants are involved in obesity-associated colon cancer (15). For instance, the FTO polymorphisms rs17817449 and rs8050136 were positively associated with obesity-associated colorectal cancer in African-Americans (11). The FTO rs9939609 variant was identified as an etiological genetic factor for obesity in Italians (16). However, no direct association of this variant with the CRC risk in the Italians was revealed by another study (7). A meta-analysis of 12 studies including 5,000 cases and 9,853 controls found that the rs9939609 variant was significantly associated with the increased risk of obesity in children and adolescents (17).

Based on the fact that the FTO rs9939609 variants have shown an association with obesity in various ethnicity and obesity is a risk factor for CRC aetiology, the objective of this study was to investigate the genetic association of FTO rs9939609 variants with the risk of developing CRC in Iranians.

## Methods

### Study Population

This study was a hospital-based case-control study conducted between January 2021 and June 2021 on a total 375 participants including 125 patients with pathologically confirmed stage 4 CRC as the case group and 250 healthy people as the control group. The CRC patients were histologically diagnosed as having primary colorectal cancer and underwent surgical operation at Firoozgar Hospital, Tehran, Iran in 2020–2021. Due to the fact that all the patients were collected from one hospital and all of them were on the same stage of the disease and underwent surgery, they were treated with the same drugs. The controls were randomly selected from a prospective cohort study among a general Iranian population. The inclusion criteria of the case group included willingness to participate in this study, confirmed histopathologic of CRC in individuals, maximum 6 months elapsed since diagnosis, and age range of 35 to 70 years. Inclusion criteria of the healthy control group included willingness to participate in this study, having no malignancy, and age range of 35 to 70 years. Socio-demographic variables including age, gender, marital status, and ethnicity were collected through a face-to-face interview. This study was approved by the ethics committee of Shahid Beheshti University of Medical Sciences, Tehran, Iran (code: IR.SBMU.CRC.REC.1398.028). All participants were explained about the details of the present study, and a written consent was obtained prior to the data collection.

### Genotyping

At the beginning of the study, 5 ml of venous blood from all individuals was collected into EDTA tubes (EDTA K3, Shandong Weigao Group Medical Polymer Co., Ltd, China). The salting out method was utilized to extract deoxyribonucleic acid (DNA) from the 200 μl of whole blood samples previously stored at −70°C. The polymerase chain reaction (PCR) was performed to amplify the extracted DNA using a PCR amplification instrument (GeneQ; Hangzhou Bioer Technology Co., Ltd., Hangzhou, China) and master mix DNA polymerase (cat. No A180301; Ampliqon, Denmark). The tetra-primer amplification refractory mutation system-polymerase chain reaction (T-ARMS-PCR) was then utilized to determine the genotype of FTO gene rs9939609 polymorphism. The sequences for the primers are presented in (**Table 1**).

**Table 1:** The features of primers used in this study.

Gene	Primer sequence	Product size	Annealing temp. °C
**FTO**	Forward outer primer: AGTTCCAGTCATTTTTGACAGC Reverse outer primer: AGCCTCTCTACCATCTTATGTC Forward inner primer: CCTTGCGACTGCTGTGAATATA Reverse inner primer: GAGACTATCCAAGTGCATCTCA	Control fragment: 429bp T allele: 278bp A allele: 194bp	59.5

### Statistical Analysis

Hardy-Weinberg equilibrium was used for evaluation of genotype distribution. General characteristics of the patients and healthy participants and the frequency of the *FTO* rs9939609 polymorphism in the case and control groups were compared by chi-square (for qualitative variables) and independent t-test (for quantitative variables) methods. Binary logistic regression analysis was performed, and regression models were fitted to investigate the association between CRC and the risk allele based on the dominant genetic model (TT *vs.* AT+AA) after adjusting for age (model 1); age and gender (model 2); age, gender, and marital status (model 3); age, gender, marital status, and ethnicity (model 4). The confounders were selected based on previous investigations (11). The statistical analyses were performed using SPSS software version 20 (SPSS Inc., Chicago, USA), and in all analyses, P-value <0.05 was considered as statistically significant.

## Results

**Figure 1:**
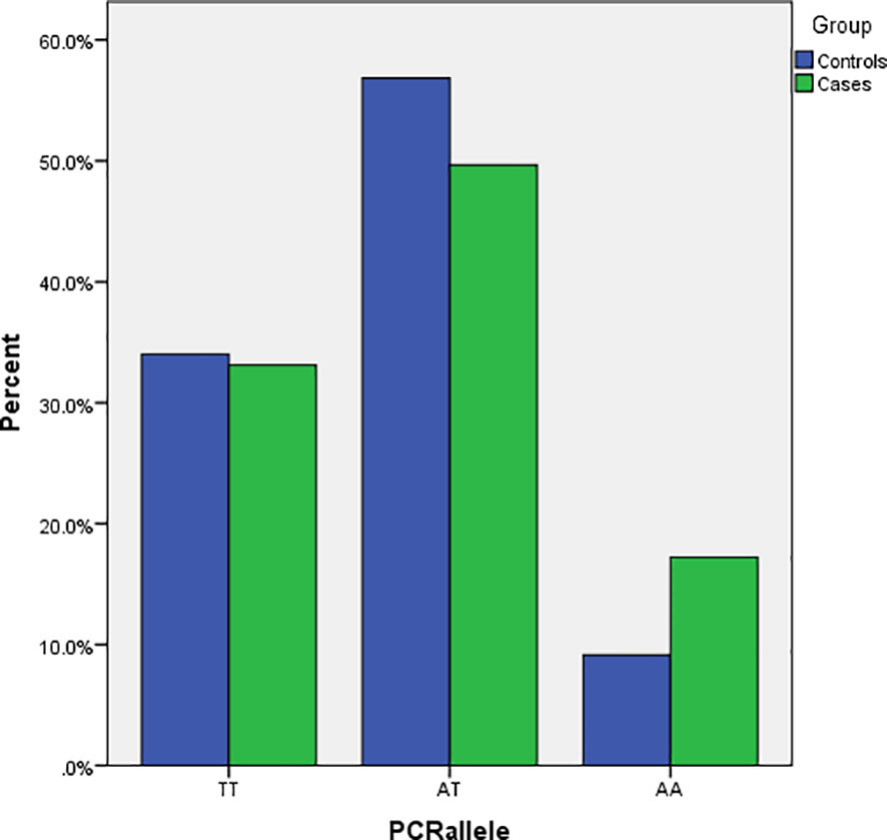
Number of risk alleles of FTO rs9939609 gene polymorphism in the case and control groups.

The general characteristics of the patients and healthy participants included in this study are presented in **Table 2**. The mean (± SD) age in the case and control groups was 53.2 (± 1.34) and 41.63 (± 1.89) years, respectively (P <0.05). Between the case and control groups, significant differences exist for the marital status (P<0.01) of the participants, with the case groups having a lower number (6.4%) of single participants compared to the control group (23.5%). There was no significant difference between the two groups in terms of gender, weight, height, and BMI. The participants represent six different ethnicity subclusters within Iranians, with no significant difference between the case *versus* control groups for any of the subclusters. The genotype frequencies in both control and pathological subjects did not deviate from the Hardy–Weinberg equilibrium (P=0.06 and 0.87, respectively). Genotyping of the *FTO* rs9939609 polymorphism revealed a significant difference (P<0.05) between the case *versus* the control groups. The AA genotype is over-represented in the cases (16.4%) than the controls (10%) (**Figure 1**).

**Table 2:** Characteristics of study population.

Characteristics	Control (*n = 250*)	Case (*n = 125*)	*P*-value
Age (y)^*^	41.63 ± 1.89	53.2 ± 1.34	<0.001^†^
Gender^‡^			
Male	32 (47.1%)	61 (55.5%)	0.276^¶^
Female	36 (52.9%)	49 (44.5%)	
Weight (kg)	70.2 ± 8.6	69.7 ± 10.9	0.74
Height (cm)	159.27 ± 8.4	156.19 ± 5.6	0.07
BMI (kg/m^2^)	27.63 ± 3.1	28.58 ± 4.1	0.08
Marital Status^‡^			
Single	58 (23.5%)	8 (6.4%)	0.003^¶^
Married	187 (75.0%)	112 (89.1%)	
Divorced	5 (1.5%)	5 (4.5%)	
Ethnicity^‡^			
Persian	183 (73.4%)	76 (61.0%)	0.528^¶^
Turkish	43 (17.2%)	33 (25.6%)	
Lor	20 (7.8%)	9 (7.3%)	
Kurdish	4 (1.6%)	5 (3.7%)	
Arab	0	1 (1.2%)	
Other	0	1 (1.2%)	
Genotype (rs9939609)^‡^			
0 (TT)	85 (34%)	45 (36.4%)	0.020^¶^
1 (AT)	140 (56%)	59 (47.3%)	
2 (AA)	25 (10%)	21 (16.4%)	
			

^*^Values are means ± SD.

^†^P-values obtained using t test.

^‡^Number of participants having the characteristic (%).

^¶^P-values obtained using Chi-square test.

The association between FTO rs9939609 polymorphism and CRC was investigated by logistic regression, and the results are presented in (**Table 3**). The association between the risk allele (A) and the incidence of CRC (B=1.67, P<0.05) remained significant after adjustment for age. Further adjustment for gender (model 2) and marital status (model 3) did not change this result (B=1.67, P= 0.042 and B=1.67, P=0.043, respectively). The effect remained significant even after additional adjustment for the ethnicity (B=1.57, P= 0.047) of the participants.

**Table 3:** Beta estimates and confidence intervals for the association between FTO rs9939609 polymorphism and CRC.

Model	Components	Chi-square	*df*	B	P-value
Model 1		21.46	*3*		*0.000*
	Genotype (TT)				0.111
	Genotype (TA)			−1.325	0.111
	Genotype (AA)			−1.666	*0.042*
	Age			0.042	*0.000*
Model 2		22.89	*5*		*0.000*
	Genotype (TT)				0.118
	Genotype (TA)			−1.380	0.099
	Genotype (AA)			−1.672	*0.042*
	Gender			−0.438	0.231
	Age			0.041	*0.001*
Model 3		25.26	*10*		0.005
	Genotype (TT)				0.124
	Genotype (TA)			−1.424	0.090
	Genotype (AA)			−1.676	*0.043*
	Gender			−0.542	0.154
	Marital (Unmarried)				0.327
	Marital (Married)			−1.190	0.422
	Marital (Unknown)			−0.213	0.869
	Age			0.029	*0.039*
Model 4		29.00	*11*		0.002
	Genotype (TT)				0.170
	Genotype (TA)			−1.269	0.143
	Genotype (AA)			−1.570	0.067
	Gender (Male)			−0.506	0.191
	Ethnicity (Fars)				0.852
	Ethnicity (Turk)			−21.295	1.000
	Ethnicity (Lor)			−20.911	1.000
	Ethnicity (Kurd)			−21.117	1.000
	Ethnicity (Arab)			−19.685	1.000
	Ethnicity (Others)			−2.263	1.000
	Marital (Unmarried)			0.313	
	Marital (Married)			−1.277	0.396
	Marital (Unknown)			−0.265	0.840
	Age			0.030	*0.038*

## Discussion

The present study investigated the genetic association of FTO rs9939609 variant with the risk of developing colorectal cancer in 375 Iranians (125 patients and 250 healthy) volunteers. The genotyping revealed that the risk allele (A) is over-represented in the patients’ group. There is a significant positive association of the A allele with the risk of developing CRC in Iranians. The association of FTO rs9939609 variants with the CRC in Iranian evident in this study further suggested the shared role of energy metabolism and obesity pathways in colorectal cancer.

The present case‐control study identified the genetic association of FTO rs9939609 variant with colorectal cancer in Iranians with the A allele over-represented in the patient group. Fat-mass- and obesity-associated (FTO) gene codes a polypeptide (13) and is predominantly expressed in the brain, skeletal muscle, and adipose tissue (8). The expression of FTO within the arcuate nucleus of the hypothalamus is necessary for regulating appetite and energy metabolism (14). An excessive expression of FTO increases food intake, leading to a positive energy balance and obesity. However, the FTO rs9939609 variant being an SNP present in the first intronic region of the transcript, the underlying molecular mechanism of this SNP is not apparent.

The FTO mediates one common pathway of obesity and cancer *via* 6-methyl adenosine (m6A)-dependent demethylase activity (18). The m6A-dependent demethylase activity enables regulating the abundance and stability of mRNA of several genes, including the genes involved in satiety and hunger (ghrelin and leptin), and mTOR, a key component of regulating mRNA translation and cell growth, thus regulating the occurrence and progression of obesity and cancer (15). The FTO protein also acts as a nutrient sensor that can play a role in cell growth and proliferation. Experimental knockdown of the FTO gene resulted in disruption of cellular energy balance (15). Numerous experiments demonstrated a vital role of obesity and excessive food intake in the complex metabolic regulation of CRC tumorigenesis (ref). Expression of adipokines, hunger and satiety hormones, dysbiosis of the intestinal microbiota, and impaired bile acid homeostasis are some of the shared mechanisms underlying tumorigenesis of CRC and metabolic syndrome (19). FTO gene expression increased after intake of macronutrients such as carbohydrates and proteins that play a key role in the tumour formation process (19).

Obesity is associated with insulin resistance and hyperinsulinemia, resulting in increased production of growth factors necessary to support tumorigenesis, leading to carcinogenesis. Oxidative stress and inflammatory processes are other complications of obesity that are also involved in carcinogenesis (12, 19). Moreover, there is evidence that the A allele of the FTO rs9939609 is significantly associated with higher serum leptin concentrations (20), even independently of adiposity and BMI (21). Meanwhile, recent studies have reported that leptin may be involved in colorectal cancer development and progression (22, 23). These evidence indicates that leptin could be a possible intermediary contributing to the association between the FTO rs9939609 polymorphism and colorectal cancer. Therefore, it is reasonable to expect that if FTO variants have a genetic association with obesity, they are also likely to have a close genetic association with CRC.

One possible mechanisms of the association between FTO with CRC is the role of obesity as a chronic inflammatory stimulus in carcinogenesis. C-reactive protein (CRP) may facilitate monocyte adhesion and macrophage infiltration in the pathological tissue and can boost angiogenesis, a key hallmark of CRC (24). A recent study reported that pharmacological inhibition of FTO dramatically attenuated cell self-renewal and reprogrammed immune response by suppressing expression of immune checkpoint genes. FTO inhibition sensitizes leukemia cells to T cell cytotoxicity and overcomes hypomethylating agent-induced immune evasion, indicating the broad potential of targeting FTO for cancer therapy (25).

Nevertheless, there are reports of associations of FTO polymorphism with the susceptibility of developing various cancer independent of obesity (26, 27). This observation suggests that body mass index and weight might be confounding factors in the association between FTO polymorphism and cancer risk. A cross-sectional study also reported that A-allele of rs9939609 polymorphism was associated with higher probability of developing inflammatory bowel disease (IBD) that was independent from the BMI status of the patients (28). At the same time, those IBD patients were reported to have the high risk for developing CRC (29). Hence, it may be plausible to expect that the association between FTO polymorphism and colorectal cancer may be independent of its association with obesity-related traits.

The rs9939609 variant is one of the extensively studied SNPs of the FTO gene. This SNP was found to have genetic association with obesity-related traits. Frayling et al. reported that 16% adults carrying AA genotype weigh 3 kg more and had a 1.67-fold greater odds of developing obesity compared the genotype without A allele. While the FTO can contribute to the development of cancer through affecting several biochemical pathways (30), *genetic association* between rs9939609 polymorphism and the risk of developing cancers was reported (31). However, to date, only a few studies investigated the association of rs9939609 variant with the risk of developing CRC (11, 32). In contrast to those two studies that have not found any association of this variant with CRC in the Caucasians and mixed ethnicity, our study identified a positive association between the A allele and increased susceptibility to CRC in the Iranian patients.

A significant association between rs9939609 variants and the risk of developing other types of cancer, such as kidney cancer, pancreatic cancer, breast cancer, and endometrial cancer, was reported (31, 33–35). In a case-control analysis with 3,601 non-Hispanic white women with endometrial carcinoma and 5,275 controls, the A allele of rs9939609 was found to be associated with an increased risk of endometrial cancer (36). On the other hand, a few studies also reported associations of the A allele of rs993609 variants with a reduced risk of cancers such as the risk of lung cancer (35), while several other studies found no relation between this variant and cancer risk (11, 32, 37).

However, this study has some limitations. First, the sample size was relatively small and the mean age of the control group was significantly different from the case group. However, the effect of age on the results was adjusted in regression models. Second, the association found between CRC and an FTO genotype in this study with retrospective design (not experimental) does not necessarily mean one factor directly caused the other. Third, the cases in this study were defined by whether they did or did not have the condition, and the results cannot confirm the association of FTO gene with different types of the disease. Fourth, some confounding variables such as diet and physical activity were not considered in this study. The association of A allele with the Iranian CRC patients reported in this study warrants further validation through genotyping larger sample size and study designs, taking into consideration the age and ethnicity of the populations (30).

## Conclusion

In conclusion, this study identified a positive association between the A allele of rs9939609 polymorphism and increased susceptibility to CRC in the Iranian subjects. Further studies addressing the functional impact of this FTO polymorphism in the human colon and rectum and understanding of the underlying mechanism of the association of FTO gene with CRC are needed.

## Data Availability Statement

The raw data supporting the conclusions of this article will be made available by the authors, if they are requested, to any qualified researcher.

## Ethics Statement

This study has been approved by the local ethics review boards at Shahid Beheshti University of Medical Sciences (IR.SBMU.CRC.REC.1398.028). The patients/participants provided their written informed consent to participate in this study.

## Author Contributions

MG, SD, BB, and SHD designed the study and were involved in the data collection, analysis, and drafting of the manuscript. MA, GA, HS, EH, EKh, SR, AH, ZA, and AJ were involved in the design of the study, analysis of the data, and critically reviewed the manuscript. All authors contributed to the article and approved the submitted version.

## Funding

Funding for this study was provided by Shahid Beheshti University of Medical Sciences, Tehran, Iran. This study is related to the project NO. 15784 from Cnacer Research Center, Shahid Beheshti University of Medical Sciences, Tehran, Iran.

## Conflict of Interest

The authors declare that the research was conducted in the absence of any commercial or financial relationships that could be construed as a potential conflict of interest.

## Publisher’s Note

All claims expressed in this article are solely those of the authors and do not necessarily represent those of their affiliated organizations, or those of the publisher, the editors and the reviewers. Any product that may be evaluated in this article, or claim that may be made by its manufacturer, is not guaranteed or endorsed by the publisher.

## References

[1] Kuipers E Grady W Lieberman D Seufferlein T Sung J Boelens P. Colorectal Cancer. Nat Rev Dis Primers (2015) 1:15065. doi: 10.1038/nrdp.2015.65 27189416 PMC4874655

[2] Mármol I Sánchez-de-Diego C Pradilla Dieste A Cerrada E Rodriguez Yoldi MJ. Colorectal Carcinoma: A General Overview and Future Perspectives in Colorectal Cancer. Int J Mol Sci (2017) 18(1):197. doi: 10.3390/ijms18010197 PMC5297828 28106826

[3] Rawla P Sunkara T Barsouk A. Epidemiology of Colorectal Cancer: Incidence, Mortality, Survival, and Risk Factors. Przeglad Gastroenterol (2019) 14(2):89. doi: 10.5114/pg.2018.81072 PMC6791134 31616522

[4] Nikbakht H-A Shokri-Shirvani J Ashrafian-Amiri H Ghaem H Jafarnia A Alijanpour S. The First Screening Program for Colorectal Cancer in the North of Iran. J Gastrointestinal Cancer (2020) 51(1):165–71. doi: 10.1007/s12029-019-00226-9 30919264

[5] Shadmani FK Ayubi E Khazaei S Sani M Hanis SM Khazaei S. Geographic Distribution of the Incidence of Colorectal Cancer in Iran: A Population-Based Study. Epidemiol Health (2017) 39:e2017020. doi: 10.4178/epih.e2017020 28774167 PMC5543296

[6] Azeem S Gillani SW Siddiqui A Jandrajupalli SB Poh V Sulaiman SAS. Diet and Colorectal Cancer Risk in Asia-A Systematic Review. Asian Pacific J Cancer Prev (2015) 16(13):5389–96. doi: 10.7314/APJCP.2015.16.13.5389 26225683

[7] Doaei S Kalantari N Izadi P Salonurmi T Jarrahi AM Rafieifar S. Interactions Between Macro-Nutrients’ Intake, FTO and IRX3 Gene Expression, and FTO Genotype in Obese and Overweight Male Adolescents. Adipocyte (2019) 8(1):386–91. doi: 10.1080/21623945.2019.1693745 PMC6948981 31771407

[8] Doaei S Kalantari N Mohammadi NK Izadi P Gholamalizadeh M Eini-Zinab H. Up-Regulation of FTO Gene Expression was Associated With Increase in Skeletal Muscle Mass in Overweight Male Adolescents. Arch Med Sci: AMS (2019) 15(5):1133. doi: 10.5114/aoms.2019.87239 31572457 PMC6764316

[9] Hull R Francies FZ Oyomno M Dlamini Z. Colorectal Cancer Genetics, Incidence and Risk Factors: In Search for Targeted Therapies. Cancer Manage Res (2020) 12:9869–82. doi: 10.2147/CMAR.S251223 PMC7553623 33116845

[10] Elangovan A Skeans J Landsman M Ali SM Elangovan AG Kaelber DC. Colorectal Cancer, Age, and Obesity-Related Comorbidities: A Large Database Study. Digestive Dis Scie (2021) 66(9):3156–63. doi: 10.1007/s10620-020-06602-x 32954457

[11] Tarabra E Actis G Fadda M De Paolis P Comandone A Coda R. The Obesity Gene and Colorectal Cancer Risk: A Population Study in Northern Italy. Eur J Internal Med (2012) 23(1):65–9. doi: 10.1016/j.ejim.2011.07.011 22153534

[12] Law PJ Timofeeva M Fernandez-Rozadilla C Broderick P Studd J Fernandez-Tajes J. Association Analyses Identify 31 New Risk Loci for Colorectal Cancer Susceptibility. Nat Commun (2019) 10(1):1–15. doi: 10.1038/s41467-019-09775-w 31089142 PMC6517433

[13] Lan N Lu Y Shuangshuang P Xi H Nie X Liu J. FTO-A Common Genetic Basis for Obesity and Cancer. Front Genet (2020) 11:1149. doi: 10.3389/fgene.2020.559138 PMC7701174 33304380

[14] Frayling TM Timpson NJ Weedon MN Zeggini E Freathy RM Lindgren CM. A Common Variant in the FTO Gene is Associated With Body Mass Index and Predisposes to Childhood and Adult Obesity. Science (2007) 316(5826):889–94. doi: 10.1126/science.1141634 PMC2646098 17434869

[15] Pitman RT Fong JT Billman P Puri N. Knockdown of the Fat Mass and Obesity Gene Disrupts Cellular Energy Balance in a Cell-Type Specific Manner. PloS One (2012) 7(6):e38444. doi: 10.1371/journal.pone.0038444 22675562 PMC3367022

[16] Merra G Gualtieri P Cioccoloni G Falco S Bigioni G Tarsitano M. FTO Rs9939609 Influence on Adipose Tissue Localization in the Italian Population. Eur Rev Med Pharmacol Sci (2020) 24(6):3223–35. doi: 10.26355/eurrev_202003_20689 32271440

[17] Quan L Wang H Tian Y Mu X Zhang Y Tao K. Association of Fat-Mass and Obesity-Associated Gene FTO Rs9939609 Polymorphism With the Risk of Obesity Among Children and Adolescents: A Meta-Analysis. Eur Rev Med Pharmacol Sci (2015) 19(4):614–23. 25753879

[18] Tung YL Yeo GS O’Rahilly S Coll AP. Obesity and FTO: Changing Focus at a Complex Locus. Cell Metab (2014) 20(5):710–8. doi: 10.1016/j.cmet.2014.09.010 25448700

[19] Doaei S Kalantari N Mohammadi NK Tabesh GA Gholamalizadeh M. Macronutrients and the FTO Gene Expression in Hypothalamus; a Systematic Review of Experimental Studies. Indian Heart J (2017) 69(2):277–81. doi: 10.1016/j.ihj.2017.01.014 PMC5414942 28460778

[20] Andreasen CH Stender-Petersen KL Mogensen MS Torekov SS Wegner L Andersen G. Low Physical Activity Accentuates the Effect of the FTO Rs9939609 Polymorphism on Body Fat Accumulation. Diabetes (2008) 57(1):95–101. doi: 10.2337/db07-0910 17942823

[21] Labayen I Ruiz J Ortega F Dalongeville J Jiménez-Pavón D Castillo M. Association Between the FTO Rs9939609 Polymorphism and Leptin in European Adolescents: A Possible Link With Energy Balance Control. The HELENA Study. Int J Obes (2011) 35(1):66–71. doi: 10.1038/ijo.2010.219 20975729

[22] Koda M Sulkowska M Kanczuga-Koda L Surmacz E Sulkowski S. Overexpression of the Obesity Hormone Leptin in Human Colorectal Cancer. J Clin Pathol (2007) 60(8):902–6. doi: 10.1136/jcp.2006.041004 PMC1994494 17660334

[23] Joshi RK Kim WJ Lee S-A. Association Between Obesity-Related Adipokines and Colorectal Cancer: A Case-Control Study and Meta-Analysis. World J Gastroenterol: WJG (2014) 20(24):7941. doi: 10.3748/wjg.v20.i24.7941 24976730 PMC4069321

[24] Solimando AG Summa SD Vacca A Ribatti D. Cancer-Associated Angiogenesis: The Endothelial Cell as a Checkpoint for Immunological Patrolling. Cancers (2020) 12(11):3380. doi: 10.3390/cancers12113380 PMC7696032 33203154

[25] Su R Dong L Li Y Gao M Han L Wunderlich M. Targeting FTO Suppresses Cancer Stem Cell Maintenance and Immune Evasion. Cancer Cell (2020) 38(1):79–96.e11. doi: 10.1016/j.ccell.2020.04.017 32531268 PMC7363590

[26] Delahanty RJ Beeghly-Fadiel A Xiang Y-B Long J Cai Q Wen W. Association of Obesity-Related Genetic Variants With Endometrial Cancer Risk: A Report From the Shanghai Endometrial Cancer Genetics Study. Am J Epidemiol (2011) 174(10):1115–26. doi: 10.1093/aje/kwr233 PMC3246689 21976109

[27] Khella MS Salem AM Abdel-Rahman O Saad AS. The Association Between the FTO Rs9939609 Variant and Malignant Pleural Mesothelioma Risk: A Case–Control Study. Genet Testing Mol Biomarkers (2018) 22(2):79–84. doi: 10.1089/gtmb.2017.0146 29260910

[28] Dragasevic S Stankovic B Kotur N Sokic-Milutinovic A Milovanovic T Lukic S. Metabolic Syndrome in Inflammatory Bowel Disease: Association With Genetic Markers of Obesity and Inflammation. Metab Syndrome Related Disord (2020) 18(1):31–8. doi: 10.1089/met.2019.0090 31750766

[29] Muller M Hansmannel F Arnone D Choukour M Ndiaye NC Kokten T. Genomic and Molecular Alterations in Human Inflammatory Bowel Disease-Associated Colorectal Cancer. United Eur Gastroenterol J (2020) 8(6):675–84. doi: 10.1177/2050640620919254 PMC7437079 32268844

[30] Hernández-Caballero ME Sierra-Ramírez JA. Single Nucleotide Polymorphisms of the FTO Gene and Cancer Risk: An Overview. Mol Biol Rep (2015) 42(3):699–704. doi: 10.1007/s11033-014-3817-y 25387436

[31] Huang X Zhao J Yang M Li M Zheng J. Association Between FTO Gene Polymorphism (Rs9939609 T/A) and Cancer Risk: A Meta-Analysis. Eur J Cancer Care (2017) 26(5):e12464. doi: 10.1111/ecc.12464 26931363

[32] Lim U Wilkens LR Monroe KR Caberto C Tiirikainen M Cheng I. Susceptibility Variants for Obesity and Colorectal Cancer Risk: The Multiethnic Cohort and PAGE Studies. Int J Cancer (2012) 131(6):E1038–E43. doi: 10.1002/ijc.27592 PMC3402643 22511254

[33] Lin Y Ueda J Yagyu K Ishii H Ueno M Egawa N. Association Between Variations in the Fat Mass and Obesity-Associated Gene and Pancreatic Cancer Risk: A Case–Control Study in Japan. BMC Cancer (2013) 13(1):1–6. doi: 10.1186/1471-2407-13-337 23835106 PMC3716552

[34] Kaklamani V Yi N Sadim M Siziopikou K Zhang K Xu Y. The Role of the Fat Mass and Obesity Associated Gene (FTO) in Breast Cancer Risk. BMC Med Genet (2011) 12(1):1–10. doi: 10.1186/1471-2350-12-52 21489227 PMC3089782

[35] Brennan P McKay J Moore L Zaridze D Mukeria A Szeszenia-Dabrowska N. Obesity and Cancer: Mendelian Randomization Approach Utilizing the FTO Genotype. Int J Epidemiol (2009) 38(4):971–5. doi: 10.1093/ije/dyp162 PMC2734066 19542184

[36] Lurie G Gaudet MM Spurdle AB Carney ME Wilkens LR Yang HP. The Obesity-Associated Polymorphisms FTO Rs9939609 and MC4R Rs17782313 and Endometrial Cancer Risk in non-Hispanic White Women. PloS One (2011) 6(2):e16756. doi: 10.1371/journal.pone.0016756 21347432 PMC3035652

[37] Yang B Thrift AP Figueiredo JC Jenkins MA Schumacher FR Conti DV. Common Variants in the Obesity-Associated Genes FTO and MC4R are Not Associated With Risk of Colorectal Cancer. Cancer Epidemiol (2016) 44:1–4. doi: 10.1016/j.canep.2016.07.003 27449576 PMC5125024

